# Effects of Whole-Body Electromyostimulation on Strength-, Sprint-, and Jump Performance in Moderately Trained Young Adults: A Mini-Meta-Analysis of Five Homogenous RCTs of Our Work Group

**DOI:** 10.3389/fphys.2019.01336

**Published:** 2019-11-08

**Authors:** Nicolas Wirtz, Ulrike Dörmann, Florian Micke, André Filipovic, Heinz Kleinöder, Lars Donath

**Affiliations:** Department of Intervention Research in Training Science, Institute of Training Science and Sport Informatics, German Sport University Cologne, Cologne, Germany

**Keywords:** WB-EMS, electrical stimulation, strength training, MVC, peak power output

## Abstract

**Background:** Whole-body electromyostimulation (WB-EMS) gained increasing interest in sports within recent years. However, few intervention studies have examined the effects of WB-EMS on trained subjects in comparison to conventional strength training.

**Objective:** The aim of the present mini-meta-analysis of 5 recently conducted and published randomized controlled WB-EMS trails of our work group was to evaluate potentially favorable effects of WB-EMS in comparison to conventional strength training.

**Methods:** We included parameter of selected leg muscle's strength and power as well as sprint and jump performance. All subjects were moderately trained athletes [>2 training sessions/week, >2 years of experience in strength training; experimental group (*n* = 58): 21.5 ± 3.3 y; 178 ± 8 cm; 74.0 ± 11 kg; control group (*n* = 54): 21.0 ± 2.3 y; 179.0 ± 9 cm; 72.6 ± 10 kg]. The following WB-EMS protocols were applied to the experimental group (EG): 2 WB-EMS sessions/week, bipolar current superimposed to dynamic exercises, 85 Hz, 350 μs, 70% of the individual pain threshold amperage. The control groups (CG) underwent the same training protocols without WB-EMS, but with external resistance.

**Results:** Five extremely homogenous studies (all studies revealed an *I*^2^ = 0%) with 112 subjects in total were analyzed with respect to lower limb strength and power in leg curl, leg extension and leg press machines, sprint—and jump performance. Negligible effects in favor of WB-EMS were found for F_max_ of leg muscle groups [SMD: 0.11 (90% CI: −0.08, 0.33), *p* = 0.73, *I*^2^ = 0%] and for CMJ [SMD: 0.01 (90% CI: −0.34, 0.33), *p* = 0.81, *I*^2^ = 0%]. Small effects, were found for linear sprint [SMD: 0.22 (90% CI: −0.15, 0.60), *p* = 0.77, *I*^2^ = 0%] in favor of the EMS-group compared to CON.

**Conclusion:** We conclude that WB-EMS is a feasible complementary training stimulus for performance enhancement. However, additional effects on strength and power indices seem to be limited and sprint and jump-performance appear to be benefiting only slightly. Longer training periods and more frequent application times and a slightly larger stimulus could be investigated in larger samples to further elucidate beneficial effects of WB-EMS on performance parameters in athletes.

## Introduction

Electromyostimulation (EMS) is a common and established method to enhance muscular strength and performance. Systematic reviews have well documented beneficial influence of locally applied EMS on strength (Delitto et al., 1989; Bax et al., 2005; Requena Sánchez et al., 2005; Paillard, 2008; Filipovic et al., 2012) and the neuromuscular parameters (Vanderthommen and Duchateau, 2007). Further studies revealed positive effects on jump and sprint capacity (Wolf et al., 1986; Brocherie et al., 2005; Herrero et al., 2006, 2010a,b; Babault et al., 2007; Maffiuletti et al., 2009; Billot et al., 2010; Voelzke et al., 2012; Filipovic et al., 2016; Wirtz et al., 2016). The reasons for the improvements using EMS are a higher number of motor units recruited during exercise with EMS compared to voluntary dynamic contractions only (Kots and Chwilon, 1971). Additionally, the activation of fast-twitch fibers at relatively low force levels plays also a relevant role (Gregory and Bickel, 2005). Most studies used the maximum pain threshold (maximum tolerated amperage) to regulate the impulse intensity (amperage) (Brocherie et al., 2005; Maffiuletti et al., 2009). However, a high level of muscle tension due to EMS limits the range of dynamic movements. Therefore, in dynamic exercise modes with superimposed EMS, the impulse intensity need to be adjusted to ensure sufficient movement. 70% of maximum pain threshold is considered practicable and might be more promising, as the subjective feeling of increased remains comfortable (Wirtz et al., 2016; Micke et al., 2018). Dynamic movements with additional EMS can also increase activation levels at different muscle length and during different contraction modes, e.g., during eccentric work phases (Westing et al., 1990). Authors hypothesized that type II muscle fibers remain active during EMS in contrast to the normal continuing de-recruitment of motor units during the eccentric phase. Therefore, the intensification of exercise by superimposed EMS can potentially induce an increase in recruitment of high-threshold motor units.

Technical innovations made EMS progress from a local stimulation to a whole-body training method where several muscle groups can be trained simultaneously through an electrode belt- and vest system (e.g., miha bodytec, Augsburg, Germany). Improved handling and simplified use led to increased recognition of whole-body-EMS (WB-EMS) training for coaches and athletes. Today WB-EMS is used in leisure sports and showed effects in both individual sports (Amaro-Gahete et al., 2018) and field sports on a high-performance level (Filipovic et al., 2016). WB-EMS enables the activation of several muscle groups simultaneously, e.g., muscle chains or agonist/antagonist during multi joint movements. This allows to train strength exercise like squats or sport specific skill exercises like jumps with superimposed WB-EMS that may support a strength transfer to more complex movements.

Strength and performance adaptations are evident for trained subjects by the use of local EMS (Filipovic et al., 2012) and for untrained subjects using WB-EMS (Kemmler et al., 2018). There is however a lack of studies including performance parameters of trained subjects. Therefore, the aim of this study is to provide evidence for the effect of training with superimposed WB-EMS on lower leg strength and power as well as sprint and jump performance in trained subjects. In this regard we conducted a mini-meta-analysis focusing on individual data of 5 recent in-house WB-EMS studies (Dörmann, 2011; Wirtz et al., 2016; Micke et al., 2018; Dörmann et al., 2019; Filipovic et al., 2019). All studies were designed to characterize the impact of superimposed WB-EMS during different exercise conditions. Exercises were designed to improve strength and power of leg muscle chain and to improve jump and sprint performance. All studies included the outcome parameter strength and power of certain leg muscle groups. Furthermore, a high standardization of EMS-adjustments characterizes all studies. Our primary hypothesis was that superimposed WB-EMS favors strength and power of lower limb muscles significantly. Our secondary hypothesis was that WB-EMS favors jumping and sprinting performance significantly.

## Materials and Methods

### Participants

One hundred and twelve male (68%) and female (32%) subjects were included into this mini-meta-analysis (*n* = 112; participants characteristics are presented in Table 1). All subjects were moderately trained doing > 2 training sessions/week on a regional to national level in sports that require sprint and/or jump performances (e.g., soccer, handball, basketball, track, and field, tennis). They were examined medically and signed a written consent about the possible risks and benefits of the study. Exclusion criteria were (a) planned absences during the whole study period, (b) any training experience in WB-EMS, (c) current training programs focusing on sprinting and jumping as well as, (d) inadequate technique in the strength exercises. In order to minimize influences of unspecific training loads, all participants were asked to refrain from any changes of their habitual physical activity behavior. Furthermore, all participants were instructed to maintain their normal dietary intake before and during the study. The study protocols were approved by the “Ethics Committee of the German Sport University Cologne” and complied with the Declaration of Helsinki “Ethical Principles for Medical Research Involving Human Subjects.”

**Table 1:** Anthropometric data (mean ± SD).

	***N* (male/female)**	**Age [years]**	**Height [cm]**	**Weight [kg]**	**BMI [kg/m^**2**^]**	**Strength training experience [years]**
EG	58 (39/19)^a^	21.5 ± 3.3	178.2 ± 7.5	74.0 ± 11.2	23.2 ± 2.5	5.4 ± 3.7
CG	54 (37/17)^b^	21.0 ± 2.3	179.0 ± 8.5	72.6 ± 9.8	22.6 ± 2.1	4.4 ± 2.9

a *Dörmann et al. (2011) (5/2); Dörmann et al. (2019) (0/10); Micke et al. (2018) (14/7); Filipovic et al. (2019) (10/0); Wirtz et al. (2016) (10/0)*.

b *Dörmann et al. (2011) (5/2); Dörmann et al. (2019) (0/11); Micke et al. (2018) (12/4); Filipovic et al. (2019) (10/0); Wirtz et al. (2016) (10/0)*.

### Study Design

The aim of the current mini-meta-analytical review was to compare the pooled favorable effects of submaximal, superimposed dynamic WB-EMS (EG) with the effects of dynamic athletic training without WB-EMS (CG) on (1) strength and power performance as well as on (2) sprinting and jumping performance. To adequately address our hypothesis we conducted individual data analysis derived from 5 randomized controlled trials (RCT) with parallel group designs (WB-EMS vs. active control) carried out between 2010 and 2017 by the Institute of Training Science and Sport Informatics, German Sport University Cologne, Germany. For the present meta-analysis, we initially selected in-house studies that (1) included trained subjects aged 18–30 years training on regional to national level and had at least 2 years of strength training experience but NO previous WB-EMS experience; (2) applied a randomized controlled trial (RCT) approach with parallel group designs (WB-EMS vs. active control); randomization by minimization method (strata: age, gender, strength training experience); (3) applied a WB-EMS protocol for 4–8 weeks with 2 training sessions per week; (4) conducted the same test settings for sprint, jump, power and strength diagnostics. Eligibility and study quality [Physiotherapy Evidence Database (PEDro) scale] were assessed (Table 2).

**Table 2:** PEDro scores and sum of the included study scores.

**References**	**Eligibility specified**	**Subjects randomly allocated**	**Concealed allocation**	**Similar baseline values**	**Blinding of subjects**	**Blinding of therapist**	**Blinding of assessor**	**Dropout <15%**	**Received treatment as allocated**	**Statistical between-group comparison**	**Point measures and variability provided**	**Sum**
Dörmann et al. (2011)	√	√	–	√	–	–	–	√	√	√	√	7
Dörmann et al. (2019)	√	√	–	√	–	–	–	√	√	√	√	7
Filipovic et al. (2019)	√	√	–	√	–	–	–	√	√	√	√	7
Micke et al. (2018)	√	√	–	√	–	–	–	√	√	√	√	7
Wirtz et al. (2016)	√	√	–	√	–	–	–	√	√	√	√	7

### Training Procedure

For detailed training procedures of the single trials, the reader is kindly referred to the corresponding studies. Studies conducted squats (Wirtz et al., 2016), squats and lunges (Dörmann et al., 2011), squat jumps (Filipovic et al., 2019) or different strength and conditioning exercises such as squats, lunges, nordic curl, skippings, heelings, lateral, horizontal, and vertical jumps (Micke et al., 2018; Dörmann et al., 2019) (Table 3). These exercises followed the recommendations to increase sprinting and jumping performance (Young, 2006; Stojanovic et al., 2017).

**Table 3:** Overview of the included studies.

**References**	**Study design**	**Sample: population; sample size (n); age, y (mean ± SD)**	**Groups**	**Intervention**	**Training characteristics**	**Outcome measures**	**Study quality (PEDro score)**
Dörmann et al. (2011)	Randomized controlled trial, two arms	Healthy sport students, *n* = 14; 21.3 ± 1.7 y	EG (*n* = 7) CG (*n* = 7)	Supervised training: (a) WB-EMS superimposed to squat exercises(b) Squat exercise with additional loads (10RM)	4 weeks, 2 sessions/week; 3 exercises/session, 3 sets, total net exercise time: 72 min	F_max_ and P_max_ at Leg Curl and Leg Press Machine; Linear Sprint time; counter movement jump height	7
Dörmann et al. (2019)	Randomized controlled trial, two arms	Healthy sport students, *n* = 21; 19.7 ± 1.7 y	EG (*n* = 10) CG (*n* = 11)	Supervised training: (a) WB-EMS superimposed to squat exercises, nordic curl, sprint and jump training(b) Squat exercise (10 RM), sprint and jump training	4 weeks, 2 sessions/week; 4–5 exercises/session, 3 sets, total net exercise time: 91 min	F_max_ and P_max_ at Leg Curl, Leg Extension and Leg Press Machine; Linear Sprint time; counter movement jump height	7
Filipovic et al. (2019)	Randomized controlled trial, two arms	Healthy sport students, *n* = 20; 24.4 ± 4.0 y	EG (*n* = 10) CG (*n* = 10)	Supervised training: (a) WB-EMS superimposed to jump training(b) Jump training	8 weeks, 2 sessions/week; 1 exercise/session, 3 sets, total net exercise time: 32 min	F_max_ and P_max_ at Leg Curl, Leg Extension, and Leg Press Machine; counter movement jump height	7
Micke et al. (2018)	Randomized controlled trial, two arms	Healthy sport students, *n* = 37; 20.8 ± 2.1 y	EG (*n* = 21) CG (*n* = 16)	Supervised training: (a) WB-EMS superimposed to squat exercises, Nordic curl, sprint and jump training(b) Squat exercises, Nordic curls, sprint and jump training	8 weeks, 2 sessions/week; 4–5 exercises/session, 3 sets, total net exercise time: 113 min	F_max_ and P_max_ at Leg Curl, Leg Extension, and Leg Press Machine; Linear Sprint time; counter movement jump height	7
Wirtz et al. (2016)	Randomized controlled trial, two arms	Healthy sport students, *n* = 20; 22.1 ± 1.9 y	EG (*n* = 10) CG (*n* = 10)	Supervised training:(a) WB-EMS superimposed to squat exercises with additional loads (10 RM)(b) Squat exercise with additional loads (10 RM)	6 weeks, 2 sessions/week; 1 exercise/session, 4 sets, total net exercise time: 48 min	F_max_ and P_max_ at Leg Curl, Leg Extension, and Leg Press Machine; Linear Sprint time; counter movement jump height	7

All studies implemented an active control group that completed the same exercise protocol without superimposed WB-EMS. All studies had a standardization procedure using the same parameters for both tested groups (EG and CG) like exercises, number of repetitions, number of sets, range of motion, movement velocity, and rate of perceived exertion. In general, all participants completed 8–16 training sessions during a 4–8 week period conducting 2 training sessions per week with a total intervention time under tension between 32 and 113 min.

All WB-EMS interventions complied with the guidelines for a safe and effective WB-EMS training (Kemmler et al., 2016). The miha bodytec system (Augsburg, Germany) was employed as EMS device (cf. Kemmler et al., 2012). The application unit was connected via electrical cords to a stimulation vest and belts. Thereby, bilaterally paired surface electrodes were integrated. 8 muscle areas could be stimulated synchronously with freely selectable impulse intensities (0–120 mA) for each pair of electrodes. In our studies, 3 paired electrodes were applied around the muscle belly of the lower legs (27 cm length × 4 cm width), the thighs (44 × 4 cm) and at the buttocks (13 × 10 cm). Additionally, the upper body was stimulated with 2 bilaterally paired electrodes integrated in the stimulation vest at the lower back (14 × 11 cm) and abdominals (23 × 10 cm).

The WB-EMS adjustments as well as the progression for the conventional strength and conditioning programs were equal for all interventions. The intensity of each exercise set was controlled by Borg Rating of Perceived Exertion (RPE) (Tiggemann et al., 2010). If a set was no longer exhaustive (RPE <16 “hard”) the intensity was raised and by increasing additional loads or the use of stiffer rubber bands and by higher amperage for EG. The WB-EMS impulse frequency was set at 85 Hz, the impulse width at 350–400 μs, the impulse type as bipolar and rectangle. The intensity of WB-EMS was adjusted to 70% of the individual pain threshold (iPT = maximum tolerated amperage, 0–120 mA). The iPT was verified separately for each pair of electrodes before each session. The participants stood with an inner knee angle of 170° while tensing their muscles voluntary. The verification of iPT began increasing current to iPT at the buttock, followed by the thigh, the lower leg, the abdominal, and the lower back. Then, the intensity was subsequently downregulated using the main controller at the WB-EMS device to an intensity of 70% to enable dynamic movements.

### Testing Procedure

#### Strength and Power Testing

Strength and power diagnostics took place on the Leg Curl (LC), the Leg Extension (LE), and the Leg Press (LP) machine (Edition-Line, gym80; Gelsenkirchen, Germany). Those were equipped with the digital measurement equipment Digimax (mechaTronic; Hamm, Germany). The software IsoTest and DynamicTest 2.0 enabled the measurement of the peak force F_max_ and the peak power P_max_ (5 kN strength sensor typ KM1506, distance sensor typ S501D, megaTron; Munich, Germany). The sensors were installed in line with the steel belt of the machines that lifts the selected weight plates.

Diagnostic procedures consisted of 3 isometric trials as well as 3 isoinertial trials on LE, LC, and LP. Isometric attempts were conducted at an inner knee angle of 120° on LE and LP as well as of 150° on LC. The instruction was to press as forcefully and as fast as possible against the fixed lever arm. This enabled the determination of knee joint angle-dependent force-time curve during explosive maximum voluntary contraction. Concerning isoinertial tests, the participants were requested to move the lever arm as forcefully and as fast as possible over the complete concentric range of motion (ROM). This enabled the examination of knee joint angle-dependent power-load curve during explosive maximum voluntary leg extension on LP, knee extension on LE or knee flexion on LC. The concentric ROM corresponded to 90–180° inner knee angles on LP and LE as well as to 170–80° inner knee angles on LC. Additional load (AL) was calculated individually as a percentage of an isometric attempt at starting position of isoinertial tests. These were 90° on LP and LE as well as 170° on LC. Three attempts were conducted with 40% AL on LE and LC as well as 3 attempts with 60% AL on LP. The rest was defined as 60s between every single trial and 3 min between the different test types. The parameters F_max_ [N] and P_max_ [W] were calculated for statistical analysis and data presentation as best performance data. Reliability was determined by the coefficient of variation (CV) and the intraclass correlation coefficient (ICC) for parameters force (F) (CV < 8%; ICC 0.95–0.97), as well as for power (P) and power factors (F·V) (CV < 9%; ICC 0.84–0.97) for all used machines (Dörmann, 2011).

#### Sprint Testing

Sprint testing involved a 20 m sprint. The test was performed with a self-initiated standing start with no hopping or backward movement prior the start. Double infrared photoelectric barriers (DLS/F03, Sportronic; Leutenbach-Nellmersbach, Germany) were used to measure the time. The best sprinting time out of 2 attempts was used for subsequent analysis. The participants had 2 min rest between the trials. Sprint running performance tests (linear and change of direction) were shown as highly relative reliable (CV 1–6%; ICC 0.80–0.96) (Green et al., 2011).

#### Jump Testing

Following one familiarization jump trial, the participants performed 3 counter movement jumps (CMJ). The participants were instructed to start jumping from an upright standing position, squatting down to a knee angle of approximately 90° in order to jump as high as possible. Hands had to remain in the akimbo position for the entire movement of each jump to minimize the influence of arm swing. The highest jump was used for subsequent analysis. The Optojump system (Microgate; Bolzano, Italy) was used to verify jump height by the flight time method. It is based on measurements of optical light emitting diodes. Optojump based jump height was shown as highly relative reliable (CV < 3% and ICC > 0.9) (Glatthorn et al., 2011).

#### Statistical Analysis

Standardized mean differences (SMD, with 90% confidence intervals) were computed for each study using the adjusted Hedges' g (Equation 1). This adjustment takes small sample biases into account. The Cochrane Review Manager Software (RevMan 5.3.5, Cochrane Collaboration, Oxford, UK) was used to compute the inverse-variance method according to Deeks and Higgins (2010). Analyses were conducted with a random effects model (Borenstein et al., 2010). Forest plots were generated for each outcome category (performance, physical performance surrogates and psychological variables). The magnitude of effect sizes was classified according to the following scale: 0–0.19 = negligible effect, 0.20–0.49 = small effect, 0.50–0.79 = moderate effect and 0.80 = large effect (Cohen, 1988).

Equation 1: Equation to calculate standardized mean differences (SMD) adjusting for small sample sizes.

(1) $$\begin{array}{l} SMD_{i}=\frac{m_{1i}-m_{2i}}{s_{i}}\left(1-\frac{3}{4N_{i}-9}\right) \end{array}$$

## Results

### Strength and Power

**Figure 1:**
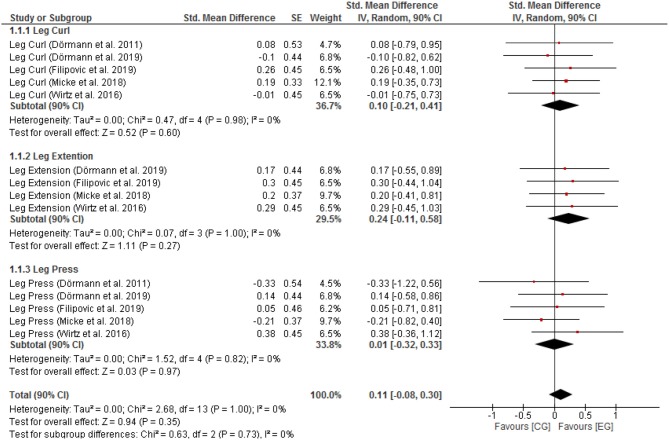
Fmax for CG vs. EG. SE, standard error; CI, confidence interval; Std., standardized; IV, independent variable.

**Figure 2:**
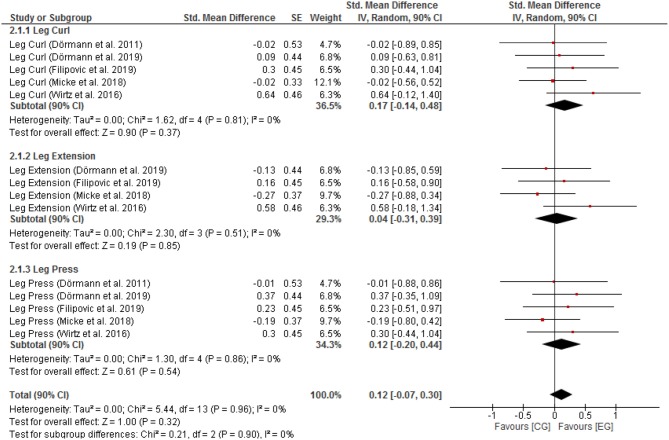
Pmax for CG vs. EG. SE, standard error; CI, confidence interval; Std., standardized; IV, independent variable.

Negligible effects with low heterogeneity were found for F_max_ leg muscle groups [SMD: 0.11 (90% CI: −0.08, 0.33), *p* = 0.73, *I*^2^ = 0%; Figure 1)]. Negligible effects with low heterogeneity were also found for P_max_ leg muscle groups [SMD: 0.12 (90% CI: −0.07, 0.30), *p* = 0.90, *I*^2^ = 0%; Figure 2].

### Linear Sprint

**Figure 3:**
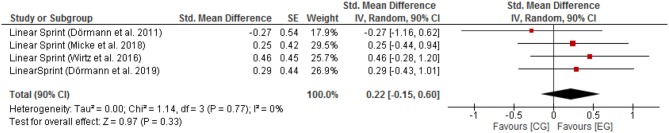
Linear sprint for CG vs. EG. SE, standard error; CI, confidence interval; Std., standardized; IV, independent variable.

Small effects with low heterogeneity were found for linear sprint [SMD: 0.22 (90% CI: −0.15, 0.60), *p* = 0.77, *I*^2^ = 0%; Figure 3] in favor of EG to CG.

### Counter Movement Jump

**Figure 4:**
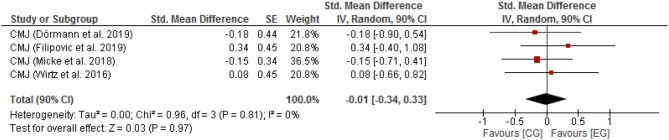
Counter Movement Jump for CG vs. EG; SE, standard error; CI, confidence interval; Std., standardized; IV, independent variable.

Neglibible effects with low heterogeneity were found for CMJ height [SMD: 0.01 (90% CI: −0.34, 0.33), *p* = 0.81, *I*^2^ = 0%; Figure 4]. Data of all analyzed parameter are summarized in supplementary material (Data Sheet 1–4).

## Discussion

This meta-analysis investigated the pooled effect sizes of superimposed WB-EMS in comparison to conventional strength and conditioning training on (1) strength and power of lower limb muscles as well as on (2) jumping and sprinting performance. Our analyses rely on findings of five homogenous studies of overall high study quality of our work group. It was hypothesized that additional superimposed WB-EMS may lead to favorable strength and power improvements of lower limb muscles as well as increases in jumping and sprinting performance compared to training without WB-EMS.

The main findings indicate that superimposed WB-EMS did not lead to superior effects of strength and power of lower limb muscle groups and jumping performance. Interestingly, sprint performance benefited with small but meaningful effects when applying WB-EMS. Generally, PEDro score analyses of the included studies revealed a “low risk of bias.” Blinding of participants and personnel is difficult in exercise studies in general and WB-EMS investigation in particular. Promoting evidence-based WB-EMS training programs requires studies that consider intention to treat analysis, blinding assessors, and reporting the risk of co-interventions. Future studies should account for these quality criteria.

Our pooled data analyses do not corroborate superior effects of WB-EMS on certain muscle groups. A small effect was merely observed for F_max_ for leg extension, but not for leg curl and leg press or LP. These results are surprising as leg extension exercise, such as squats were included in each intervention in both groups (EG and CG). The application of superimposed WB-EMS might beneficially affect maximal strength of the quadriceps muscles when additional motor unit recruitment with (a) a continuous and exhausting contractile activity in the same pool of motor units during the entire exercise period, (b) a supramaximal temporal recruitment imposed by the high stimulation frequency chosen, and (c) a synchronous recruitment of neighboring muscle fibers might account for these strength gains (Requena Sánchez et al., 2005) is guaranteed. Nevertheless, the overall effects are close to the zero-line of the forest plot and it would be speculative to assume that a higher sample sizes or longer application time lead to differential results. However, one study showed muscle group-specific adaptations of hamstring power after squats with superimposed WB-EMS (Wirtz et al., 2016). Despite notable co-activation (Zink et al., 2001), it has been reported that the activation of hamstring muscles is not affected by additional loading during squat exercise (Nuzzo and McBride, 2013). EMS can lead to higher hamstring muscle activation during lengthening and shortening, which would then results in higher hamstring maximum force in the WB-EMS training group. One potential explanation for these inconsistent results could be that the different underlying exercises and populations of the included studies, such as squats (Wirtz et al., 2016), jumps (Filipovic et al., 2019) or different strength and conditioning hamstring exercises (Micke et al., 2018) increase variability of the occurrence and magnitude of potential effects. Available evidence on the adaptability of the hamstrings employing WB-EMS independent from the type of exercise is needed and would provide specific insights into WB-EMS use to strengthen the hamstrings and to possibly prevent injuries. Particularly hamstring strains are reported to be the most prevalent muscle injury in various team and sprint related sports (Goode et al., 2015).

The simultaneous activation of (1) multiple muscle groups and (2) agonistically and antagonistically working muscles through WB-EMS combined with strength and training exercises was repeatedly reported to have the potential to improve sport-specific skills such as sprinting and jumping. Although WB-EMS triggers a seemingly counterproductive firing of the agonist and antagonist, a voluntary contraction reduces relative co-activation of antagonistic muscles, in order to continue the required dynamic exercise. Indeed, available data do not suggest such effects during superimposed WB-EMS for jumping performance. Thus, it might be beneficial to focus on specific jump exercise with superimposed EMS (Filipovic et al., 2019). In line with this reasoning, one study with professional soccer players revealed that jumps with superimposed WB-EMS in addition to soccer training sessions can be effective for jump improvements (Filipovic et al., 2016). Overall, it appears to be plausible to assume that exercise specificity and the training status of the participants affect the effects of superimposed WB-EMS. Potential improvements by the use of maximal and locally applied EMS also rely on training specificity, such as combined plyometric training (Herrero et al., 2006) and sport specificity on a higher level in sports with numerous jumps like volleyball (Malatesta et al., 2003; Voelzke et al., 2012) or basketball (Maffiuletti et al., 2000). For sprinting performance, however, minor effects could be found. The results of linear sprint are in accordance with studies that applied isometric local EMS with additional separately performed strength and sprint training sessions: One study reported improvements of 10 m skating time for 2^nd^ league ice hockey players, who trained on ice parallel to training intervention, what could elicited utilization effects (Brocherie et al., 2005). It is however reasonable that effects of WB-EMS on sprinting performance as well as jumping performance could depend on athletes' training status.

However, some limitations of the present study need to be addressed that should be considered for further research on WB-EMS. One is seen in the small sample sizes of each included studies. However, outcomes and assessment over all studies are very homogenous. Although we intended to increase the cumulative power by pooling the data, a compiled and robust effect cannot be found. Although minor improvement in top level athletes can be considered relevant, negligible additional benefits from WB-EMS in comparison to conventional training can be summarized. Ultimately, WB-EMS provides a variety of different training stimuli but a notable transfer of the results to top level athletes is speculative. Furthermore, the lack of data after a detraining phase hamper an identification of delayed effects that could potentially occur. The adaptations of WB-EMS over time need to be further investigated and concepts for periodization in high performance sports also including WB-EMS need to be developed. A further limitation is seen in the inclusion of both genders. Although Maffiuletti et al. (2008) demonstrated that supra-motor thresholds were significantly lower in women than in men, contrary to the expected constitutional differences like subcutaneous fat thickness, women showed no significant differences at motor threshold. However, the subjective tolerance to current intensity remains a key limiting factor of WB-EMS, regardless of sexes (Gregory and Bickel, 2005). Nevertheless, we did not focus on sex differences in trainability. Further limitations are seen in the different designs for lower limb exercises in the studies and the different intervention duration (4–8 weeks) that result in 8–16 total training sessions. However, 4–8 weeks can be regarded as a common and reasonable meso-cycle within periodization considerations.

Only few drop-outs occurred independent of the WB-EMS intervention and an attendance rate of 100% for all of the 112 included participants was observed and no adverse event was reported. Current intensities around 70% of maximum pain threshold that enables movement complied with the safety recommendations published by Kemmler et al. (2016). This is particularly important with regard to cases of rhabdomyolysis after WB-EMS training at maximum intensity with professional soccer players (Kastner et al., 2015).

Finally, we can conclude that WB-EMS is a feasible complementary training stimulus for performance enhancement. Additional effects of WB-EMS on relevant strength, power and performance indices seem limited. Longer training periods and more frequent application times and a slightly larger stimulus could be investigated in larger studies in order to further elucidate beneficial effects of WB-EMS on crucial performance parameters in athletes.

## Data Availability Statement

The raw data supporting the conclusions of this manuscript will be made available by the authors, without undue reservation, to any qualified researcher.

## Author Contributions

UD, NW, FM, AF, and HK conceived and designed research. UD, NW, FM, and AF conducted experiments. UD, LD, and NW analyzed data. NW, LD, and UD wrote the manuscript. HK, UD, NW, FM, AF, and LD revised the manuscript. All authors read and approved the manuscript.

### Conflict of Interest

The authors declare that the research was conducted in the absence of any commercial or financial relationships that could be construed as a potential conflict of interest.

## Supplementary Material

Data Sheet 1 Fmax pre-post in Leg Curl (LC), Leg Extension (LE), and Leg Press (LP) for CG and EG (mean, standard deviation, difference pre-post in %, effect sizes pre-post and standard error).

Data Sheet 2 Pmax pre-post in Leg Curl (LC), Leg Extension (LE), and Leg Press (LP) for CG and EG (mean, standard deviation, difference pre-post in %, effect sizes pre-post and standard error).

Data Sheet 3 Sprint time 20 m pre-post for CG and EG (mean, standard deviation, difference pre-post in %, effect sizes pre-post and standard error).

Data Sheet 4 Jump high pre-post for CG and EG (mean, standard deviation, difference pre-post in %, effect sizes pre-post and standard error).
