# Supplementary material for: Effects of Whole-Body Electromyostimulation on Strength-, Sprint-, and Jump Performance in Moderately Trained Young Adults: A Mini-Meta-Analysis of Five Homogenous RCTs of Our Work Group
Source: Front Physiol. 2019 Nov 8;10:1336. doi: 10.3389/fphys.2019.01336 (PMC6857204; doi:10.3389/fphys.2019.01336)
Supplement: Data Sheet 1 — Fmax pre-post in Leg Curl (LC), Leg Extension (LE), and Leg Press (LP) for CG and EG (mean, standard deviation, difference pre-post in %, effect sizes pre-post and standard error). [file Data_Sheet_1.PDF]

Study	Parameter	n EG	mean_pre EG [N]	SD_pre EG [N]	mean_post EG [N]	SD_post EG [N]	n CG	mean_pre CG [N]	SD_pre CG [N]	mean_post CG [N]	SD_post CG [N]	Difference Δpre-post between EG-CG [%]	**effect size**	**standard error**
Dörmann et al. 2011	Fmax LC	7	1263,7	329,8	1315,3	321,2	7	1353,4	328,0	1377,3	382,1	2,3	**0,08**	**0,53**
Dörmann et al. 2019	Fmax LC	10	726,4	121,8	842,7	186,9	11	722,2	242,6	858,8	160,6	-2,9	**-0,10**	**0,44**
Filipovic et al. 2019	Fmax LC	21	1102,4	279,0	1203,1	285,2	16	1150,9	343,5	1192,2	331,8	5,5	**0,19**	**0,33**
Micke et al. 2018	Fmax LC	10	1503,4	179,5	1593,3	221,5	10	1226,3	263,6	1317,8	207,5	-1,5	**-0,01**	**0,45**
Wirtz et al. 2016	Fmax LC	10	1455,7	173,6	1585,0	248,7	10	1366,4	236,6	1439,1	174,4	3,6	**0,26**	**0,45**
Dörmann et al. 2011	Fmax LE	0					0							
Dörmann et al. 2019	Fmax LE	10	1495,5	208,7	1657,1	348,3	11	1444,7	255,3	1565,5	276,9	2,4	**0,17**	**0,44**
Filipovic et al. 2019	Fmax LE	17	2210,7	566,4	2344,2	577,4	13	2186,4	569,7	2203,8	520,4	5,2	**0,20**	**0,37**
Micke et al. 2018	Fmax LE	10	2534,8	363,2	2569,5	443,0	10	2120,9	405,4	2039,9	332	5,2	**0,29**	**0,45**
Wirtz et al. 2016	Fmax LE	10	2405,3	331,7	2509,0	310,2	10	2379,6	342,8	2378,4	316,2	4,4	**0,30**	**0,45**
Dörmann et al. 2011	Fmax LP	7	3019,6	1400,9	3352,6	1465,8	7	3376,6	894,8	4130,1	987,6	-11,3	**-0,33**	**0,54**
Dörmann et al. 2019	Fmax LP	10	2873,0	585,0	3087,3	744,2	11	2615,5	656,4	2737,2	666,7	2,8	**0,14**	**0,44**
Filipovic et al. 2019	Fmax LP	17	2890,0	809,4	3135,5	789,1	13	2728,9	584,4	3132,2	703,7	-6,3	**-0,21**	**0,37**
Micke et al. 2018	Fmax LP	10	4212,3	471,4	5257,5	1018,7	10	3745,6	798,7	4532,1	1286,4	3,8	**0,38**	**0,45**
Wirtz et al. 2016	Fmax LP	9	3390,7	888,7	3866,0	950,3	10	3263,8	916,8	3695,0	1126,2	0,8	**0,05**	**0,46**
