# Supplementary material for: Effects of Whole-Body Electromyostimulation on Strength-, Sprint-, and Jump Performance in Moderately Trained Young Adults: A Mini-Meta-Analysis of Five Homogenous RCTs of Our Work Group
Source: Front Physiol. 2019 Nov 8;10:1336. doi: 10.3389/fphys.2019.01336 (PMC6857204; doi:10.3389/fphys.2019.01336)
Supplement: Data Sheet 2 — Pmax pre-post in Leg Curl (LC), Leg Extension (LE), and Leg Press (LP) for CG and EG (mean, standard deviation, difference pre-post in %, effect sizes pre-post and standard error). [file Data_Sheet_2.PDF]

Study	Parameter	n EG	mean_pre EG [W]	SD_pre EG [W]	mean_post EG [W]	SD_post EG [W]	n CG	mean_pre CG [W]	SD_pre CG [W]	mean_post CG [W]	SD_post CG [W]	Difference Δpre-post between EG-CG [%]	**effect size**	**standard error**
Dörmann et al. 2011	Pmax LC	7	652,9	211,0	673,4	189,5	7	683,4	207,4	708,0	229,9	-0,5	**-0,02**	**0,53**
Dörmann et al. 2019	Pmax LC	10	334,4	84,6	400,2	105,8	11	332,8	106,7	389,6	101,9	2,6	**0,09**	**0,44**
Filipovic et al. 2019	Pmax LC	21	552,7	215,7	617,6	184,8	16	565,3	168,9	635,2	182,4	-0,6	**-0,02**	**0,33**
Micke et al. 2018	Pmax LC	10	822,1	122,3	917,2	169,5	10	708,6	156,9	709,2	137,2	11,5	**0,64**	**0,46**
Wirtz et al. 2016	Pmax LC	10	817,0	99,5	843,2	117,0	10	767,2	168,1	750,0	138,4	5,4	**0,30**	**0,45**
Dörmann et al. 2011	Pmax LE	0					0							
Dörmann et al. 2019	Pmax LE	10	697,2	156,3	767,6	180,5	11	717,6	192,8	812,2	198,6	-3,1	**-0,13**	**0,44**
Filipovic et al. 2019	Pmax LE	17	1094,5	335,3	1132,1	334,6	13	1000,8	293,0	1127,8	313,1	-9,3	**-0,27**	**0,37**
Micke et al. 2018	Pmax LE	10	1426,9	274,4	1522,8	269,7	10	1284,3	256,0	1217,3	196,3	11,9	**0,58**	**0,46**
Wirtz et al. 2016	Pmax LE	10	1252,0	199,3	1318,0	216,8	10	1262,1	283,0	1286,5	247,4	3,3	**0,16**	**0,45**
Dörmann et al. 2011	Pmax LP	7	1055,4	412,0	1186,0	364,6	7	1075,1	214,7	1208,4	198,5	0,0	**-0,01**	**0,53**
Dörmann et al. 2019	Pmax LP	10	735,1	168,1	837,0	147,9	11	819,6	250,2	837,3	231,0	11,7	**0,37**	**0,44**
Filipovic et al. 2019	Pmax LP	17	1370,8	424,1	1421,8	435,0	13	1236,8	343,6	1362,7	342,4	-6,5	**-0,19**	**0,37**
Micke et al. 2018	Pmax LP	10	1766,8	389,7	1880,3	429,7	10	1534,1	370,0	1529,6	360,0	6,7	**0,30**	**0,45**
Wirtz et al. 2016	Pmax LP	10	1467,0	254,3	1568,2	231,3	10	1453,5	324,9	1484,0	304,2	4,8	**0,23**	**0,45**
