# Supplementary material for: Effects of Whole-Body Electromyostimulation on Strength-, Sprint-, and Jump Performance in Moderately Trained Young Adults: A Mini-Meta-Analysis of Five Homogenous RCTs of Our Work Group
Source: Front Physiol. 2019 Nov 8;10:1336. doi: 10.3389/fphys.2019.01336 (PMC6857204; doi:10.3389/fphys.2019.01336)
Supplement: Data Sheet 3 — Sprint time 20 m pre-post for CG and EG (mean, standard deviation, difference pre-post in %, effect sizes pre-post and standard error). [file Data_Sheet_3.PDF]

Study	Parameter	n EG	mean_pre EG [s]	SD_pre EG [s]	mean_post EG [s]	SD_post EG [s]	n CG	mean_pre CG [s]	SD_pre CG [s]	mean_post CG [s]	SD_post CG [s]	Difference Δpre-post between EG-CG [%]	**effect size**	**standard error**
Dörmann et al. 2011	Linear Sprint	7	3,2	0,2	3,2	0,3	7	3,0	0,2	3,1	0,22	-2,0	**-0,27**	**0,54**
Dörmann et al. 2019	Linear Sprint	10	3,4	0,1	3,4	0,1	11	3,4	0,1	3,3	0,15	1,2	**0,29**	**0,44**
Filipovic et al. 2019	Linear Sprint	12	3,2	0,3	3,2	0,2	11	3,1	0,2	3,1	0,18	1,9	**0,25**	**0,42**
Micke et al. 2018	Linear Sprint	10	3,0	0,1	3,1	0,1	10	3,1	0,1	3,1	0,12	1,6	**0,46**	**0,45**
Wirtz et al. 2016	Linear Sprint	0					0							
