# Supplementary material for: Effects of Whole-Body Electromyostimulation on Strength-, Sprint-, and Jump Performance in Moderately Trained Young Adults: A Mini-Meta-Analysis of Five Homogenous RCTs of Our Work Group
Source: Front Physiol. 2019 Nov 8;10:1336. doi: 10.3389/fphys.2019.01336 (PMC6857204; doi:10.3389/fphys.2019.01336)
Supplement: Data Sheet 4 — Jump high pre-post for CG and EG (mean, standard deviation, difference pre-post in %, effect sizes pre-post and standard error). [file Data_Sheet_4.PDF]

Study	Parameter	n EG	mean_pre EG [cm]	SD_pre EG [cm]	mean_post EG [cm]	SD_post EG [cm]	n CG	mean_pre CG [cm]	SD_pre CG [cm]	mean_post CG [cm]	SD_post CG [cm]	Difference Δpre-post between EG-CG [%]	**effect size**	**standard error**
Dörmann et al. 2011	CMJ	0					0							
Dörmann et al. 2019	CMJ	10	28,0	3,4	28,9	3,3	11	30,5	3,9	32,1	4,7	-2,0	**-0,18**	**0,44**
Filipovic et al. 2019	CMJ	21	36,6	8,5	37,2	6,8	15	36,2	6,7	38,0	7,0	-3,3	**-0,15**	**0,34**
Micke et al. 2018	CMJ	10	38,9	3,2	40,4	3,9	10	39,3	5,9	40,4	3,9	1,1	**0,08**	**0,45**
Wirtz et al. 2016	CMJ	10	36,5	4,0	39,3	4,4	10	37,9	4,5	39,2	5,4	4,2	**0,34**	**0,45**
